# Irreversible covalent Bruton’s tyrosine kinase inhibitor, TAS5315 versus placebo in rheumatoid arthritis patients with inadequate response to methotrexate: a randomised, double-blind, phase IIa trial

**DOI:** 10.1136/ard-2022-223759

**Published:** 2023-05-22

**Authors:** Tsutomu Takeuchi, Sakae Tanaka, Mitsuru Murata, Yoshiya Tanaka

**Affiliations:** 1 Division of Rheumatology, Department of Internal Medicine, Keio University School of Medicine, Tokyo, Japan; 2 Saitama Medical University, Saitama, Japan; 3 Department of Orthopaedic Surgery, The University of Tokyo, Tokyo, Japan; 4 Department of Laboratory Medicine, Keio University School of Medicine, Tokyo, Japan; 5 Clinical Research Center for Medicine, International University of Health and Welfare, Tokyo, Japan; 6 The First Department of Internal Medicine, School of Medicine, University of Occupational and Environmental Health, Kitakyushu, Japan

**Keywords:** Antirheumatic Agents, Arthritis, Rheumatoid, Autoantibodies, B-Lymphocytes, Rheumatoid Factor

## Abstract

**Objective:**

To examine the efficacy and safety of TAS5315, an irreversible covalent Bruton’s tyrosine kinase inhibitor, in Japanese patients with rheumatoid arthritis (RA) refractory to methotrexate.

**Methods:**

In part A of this phase IIa double-blind study, patients were randomised to TAS5315 4 or 2 mg or placebo once daily for 12 weeks; in part B, all patients received TAS5315 for another 24 weeks. The proportion of patients meeting American College of Rheumatology criteria for 20% improvement (ACR20) at week 12 was assessed (primary endpoint).

**Results:**

Ninety-one patients were randomised in part A, and 84 entered part B. At week 12, 78.9% of patients achieved ACR20 in the TAS5315 combined group vs 60.0% with placebo (p=0.053), 33.3% vs 13.3% achieved ACR50 (p=0.072) and 7.0% vs 0.0% achieved ACR70 (p=0.294), respectively. More patients receiving TAS5315 than placebo had low disease activity or remission at week 12. Clinical and biomarker improvements were maintained during part B. Adverse event (AE) incidence in TAS5315 was similar to placebo in part A; common AEs with TAS5315 were nasopharyngitis (10.3%), pruritus (6.9%) and cystitis (5.2%). Over 36 weeks, nine patients experienced bleeding events of whom four and two patients recovered with drug continuation and interruption, respectively. Three patients recovered after TAS5315 discontinuation.

**Conclusions:**

The primary endpoint was not achieved. TAS5315 appears to have some bleeding risks, but nevertheless demonstrated numerical differences, compared with placebo, in the improvement rates of all measures of RA disease activity. Future analysis of the risk-benefit of TAS5315 should be considered.

**Trial registration numbers:**

NCT03605251, JapicCTI-184020, jRCT2080223962.

**WHAT IS ALREADY KNOWN ON THIS TOPIC**

- Biological disease-modifying antirheumatic drugs are established components of the treatment of rheumatoid arthritis (RA); however, they are not effective in all patients and can be associated with significant adverse events.
- Therefore, an unmet need is a treatment for RA at least as effective as current biologicals, but with an improved safety profile.
- TAS5315 is a Bruton’s tyrosine kinase inhibitor under development for RA.

**WHAT THIS STUDY ADDS**

- This randomised, double-blind, phase IIa study found that TAS5315 numerically improved measures of disease activity in Japanese patients with RA who had an inadequate response to methotrexate.
- Bleeding events (haemorrhage subcutaneous, petechiae, purpura, cerebellar haemorrhage and epistaxis) occurred in three patients in the TAS5315 combined group in part A and in six patients in part B; four and two patients recovered with continued drug administration and interruption, respectively, and three patients recovered after TAS5315 discontinuation.

**HOW THIS STUDY MIGHT AFFECT RESEARCH, PRACTICE OR POLICY**

- This study in patients with moderate-to-severe active RA refractory to methotrexate treatment showed that TAS5315 may have some therapeutic benefit for patients who can tolerate bleeding; further analysis of the risk-benefit of TAS5315 for RA should be considered.

## Introduction

Rheumatoid arthritis (RA) is generally characterised by persistent synovitis, leading to joint destruction and disability, affecting approximately 1 in 200 adults worldwide.^1^ Patients with RA are usually initially treated with the synthetic disease-modifying antirheumatic drug (DMARD) methotrexate (MTX), followed by combination therapy with biological DMARDs (eg, tumour necrosis factor (TNF) inhibitors) or Janus kinase (JAK) inhibitors.^2^ Many patients with RA achieve remission or low disease activity after addition of biological therapy (~75% and ~30% of patients achieve remission according to the 28-joint Disease Activity Score (DAS28) and American College of Rheumatology (ACR)/European League Against Rheumatism (EULAR) classifications, respectively).^2–4^ While currently available synthetic and biological DMARDs have significantly improved outcomes and reduced disease progression in patients with RA, several issues still need to be addressed. Not all patients achieve remission or low disease activity during treatment with biological DMARDs or JAK inhibitors.^5–9^ In addition, these treatments are associated with an increased risk of infections, such as pneumonia, sepsis and opportunistic infections. Furthermore, a postmarketing surveillance study in patients with active RA recently reported that the risk of major adverse cardiovascular events, malignancy, thrombosis and death with tofacitnib was numerically higher compared with TNF inhibitors.^10^ This observation has led to warnings regarding the use of JAK inhibitors from the United States Food and Drug Administration and the European Medicines Agency.^11 12^ Therefore, there is an unmet need for a new treatment option with a different mechanism of action in RA.

Bruton’s tyrosine kinase (BTK) is a candidate among the potential therapeutic targets for RA. BTK belongs to the Tec family and is expressed in B cells, monocytes/macrophages, mast cells, platelets and osteoclasts.^13–15^ Several molecules targeting BTK have been developed to date. TAS5315 (Taiho Pharmaceutical, Tokyo, Japan) is an irreversible, covalent BTK inhibitor that suppresses B-cell activation, as well as expression of cytokines in macrophages, such as TNF-α, and thus is expected to suppress joint inflammation.^16 17^ In addition, TAS5315 selectively inhibits BTK enzyme activity in osteoclasts by suppressing the anti-IgM-induced phosphorylation of the tyrosine kinase. Osteoclasts populate the interface between inflammatory synovial tissue and the perarticular bone surface and are involved in bone destruction.^16 17^ This suggests that TAS5315 may be able to slow the progression of bone destruction in RA. The current phase II study aimed to examine the efficacy and safety of TAS5315 in Japanese patients with RA who had previously responded inadequately to MTX.

## Methods

### Study design

**Figure 1:**
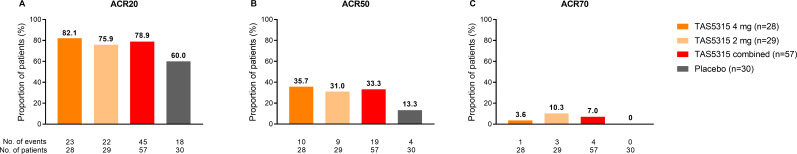
Proportion of patients achieving (A) 20% improvement in American College of Rheumatology (ACR) criteria (ACR20) at week 12 (primary endpoint), (B) ACR50 and (C) ACR70.

This was a multicentre, double-blind, placebo-controlled, randomised, parallel-group study conducted at 47 sites in Japan (NCT03605251, JapicCTI-184020). The study consisted of two parts: part A, in which patients received TAS5315 4 or 2 mg or placebo for 12 weeks; and part B, in which patients in the TAS5315 groups continued at the same dose, and those in the placebo group were switched to TAS5315 4 or 2 mg, for another 24 weeks (online supplemental methods and figure 1).

10.1136/ard-2022-223759.supp1 Supplementary data

The primary objective was to examine the efficacy of TAS5315 in combination with MTX after 12 weeks of treatment in patients with active RA who had inadequate responses to MTX.

### Patients

Eligible patients were aged ≥20 and <65 years, had RA according to the 2010 ACR/EULAR classification criteria,^18^ had inadequate response to maximally tolerated MTX doses (≥6 mg/week), moderate-to-severe RA activity (≥6/68 tender joints and ≥6/66 swollen joints), high-sensitivity C-reactive protein (hsCRP) levels of ≥0.6 mg/dL at enrolment, at least one bone erosion in the joints, and a positive rheumatoid factor (RF) test (LZ Test ‘Eiken’ RF; Eiken Chemical, Japan; cut-off >15 IU/mL) or a positive anticyclic citrullinated peptide (anti-CCP) antibody (anticitrullinated protein antibodies, ACPA) test (STACIA MEBLux CCP test; Medical and Biological Laboratories, Japan; cut-off ≥4.5 U/mL).

Key exclusion criteria are outlined in online supplemental table 1.

### Study drug

TAS5315 selectively inhibited BTK enzyme activity and suppressed anti-IgM antibody induced phosphorylation of BTK in Ramos cells.^16^ Furthermore, TAS5315 dose-dependently inhibited TNF-α production by macrophages and bone resorption activity by osteoclasts.^14 15^ In a mouse collagen-induced arthritis (CIA) model, TAS5315 dose-dependently decreased the clinical score in arthritic mice compared with that in vehicle-treated mice.^14^ In the histopathological analysis of the mouse CIA model, TAS5315 dose-dependently decreased the histopathological scores for inflammation, pannus, cartilage damage and bone damage.^14^

Phase 1 studies showed TAS5315 was tolerable when administered as repeated doses of up to 8 mg once daily for 7 days, although inhibition of platelet aggregation was observed and a small proportion of individuals developed mild subcutaneous haemorrhage and petechiae.^19^ The BTK occupancy rate was almost 100% 6 hours after administration at a TAS5315 dose of 2–8 mg.^19^

### Treatment

Patients were randomised by the central registration method using an interactive web response system in a 1:1:1 ratio to receive either TAS5315 4 or 2 mg or placebo orally once daily for the study duration, taken in combination with MTX. MTX was purchased by the participating medical institutions and provided to the patients; all expenses were covered by Taiho. TAS5315 1 mg tablets were used; patients in the 4 mg group took four TAS5315 tablets and those in the 2 mg group took two TAS5315 and two placebo tablets.

### Assessments and outcomes

Patients were followed up at weeks 1, 2 and 4, and every 4 weeks thereafter until week 36 (online supplemental figure 1). At each visit, clinical assessments, patient questionnaires, collection of adverse events (AEs) and laboratory tests were performed. X-rays of the hands and feet were taken at randomisation, week 12 and week 36. To ensure that the plain X-ray images taken across all participating institutions were consistent and could be judged accurately, investigators ensured: (1) the images included the left and right hands (including wrist joints) and feet; imgaing of each hand and one leg was required; (2) shooting parameters such as tube voltage, tube current and irradiation time were left to the discretion of the facility; (3) output (trimmed) size: 8×10 inch or 10×12 inch; (4) shooting distance: source image receptor distance was set to 100 cm; (5) focus size: small focus; (6) for the foot, shading had to be adjusted so that the region from the distal phalanx to the metatarsophalangeal joint were clearly depicted, rather than depicting the tarsal bone region; (7) the same subject was to be photographed with the same imaging conditions and position as their screening; images at screening were to be used as the reference image for comparisons.

The primary endpoint was the proportion of patients meeting ACR20 criteria at week 12 in the combined TAS5315 2 and 4 mg group versus placebo. ACR20 was defined as a 20% improvement in the tender joint count and swollen joint count (SJC) scores and a 20% improvement in three of the following five criteria: patient global assessment, physician global assessment, Health Assessment Questionnaire-Disability Index (HAQ-DI), pain Visual Analogue Scale (VAS), and erythrocyte sedimentation rate (ESR) or CRP level.^20^ Secondary endpoints were additional ACR reponse rates and other measures of disease activity, biomarkers and indicators of joint structure and bone metabolism in each of the TAS5315 2 and 4 mg groups and the combined TAS5315 group vs placebo (see online supplemental methods and table 2).

Safety was assessed by recording AEs, serious AEs (SAEs) and laboratory parameters. Bleeding-related AEs, such as haemorrhage, petechiae and ecchymosis, were considered to be AEs of special interest.

### Patient and public involvement

Patients and the public were not involved in the development of the study design or protocol, or in the recruitement for or conduct of the study, nor were they involved in the decisions to disseminate the study results. Patient-reported outcomes (patient assessment of disease activity and pain using a VAS and the HAQ-DI) were included as measures of treatment efficacy.

### Statistical analysis

Assuming that TAS5315 and placebo would have a similar effect on ACR20 response at week 12 as baricitinib and placebo did in Japanese RA patients in the RA-BEAM study,^21^ we estimated that a population of 75 patients would have 80% power to show a significant difference between TAS5315 and placebo in the primary endpoint, at a one-sided significance level of 5%. The per-protocol set (PPS) included all patients in the full analysis set (FAS) who continued to meet study eligibility criteria, were ≥80% compliant with treatment in part A and did not have any protocol violations. Missing data were imputed using non-responder imputation for categorical variables and last observation carried forward for continuous variables. Additionally, the linear extrapolation method and multiple imputation were used for the van der Heijde modification of the total Sharp Score (mTSS), bone erosion and joint space narrowing scores. Further details regarding the statistical analysis are provided in online supplemental methods.

## Results

### Patient disposition

Between 30 August 2018 and 28 May 2020, 91 patients were enrolled and randomised in part A of the study (TAS5315 4 mg: n=29; TAS5315 2 mg: n=29; placebo: n=33; online supplemental figure 2), of whom 87 patients completed the first 12 weeks of the study. Of the 91 treated patients in part A, 87 patients were included in the PPS.

Overall, 84 patients entered part B: 27 patients continued TAS5315 4 mg, 29 patients continued TAS5315 2 mg and 28 patients in the placebo group were randomised to TAS5315 4 mg (n=15) or 2 mg (n=13).

Baseline characteristics are summarised in table 1.

**Table 1:** Demographic and baseline clinical characteristics of patients (per-protocol set)

Characteristic*	TAS5315			Placebo (n=30)
	4 mg (n=28)	2 mg (n=29)	Combined (n=57)	
Female gender, n (%)	22 (78.6)	20 (69.0)	42 (73.7)	23 (76.7)
Age, years	52.0±10.6	51.9±9.1	51.9±9.8	53.5±7.2
Body weight, kg	61.09±12.12	61.38±12.12	61.24±12.01	60.36±13.76
BMI, kg/m^2^	23.92±3.75	23.88±5.26	23.90±4.54	23.41±5.53
Duration of RA, days	1936.1±1529.0	2038.7±1712.0	1988.3±1611.0	2259.0±1775.4
Oral corticosteroid use (yes), n (%)	12 (42.9)	10 (34.5)	22 (38.6)	13 (43.3)
Prednisolone-equivalent dose, mg/day	3.8±1.9	4.1±2.5	3.9±2.1	3.7±1.8
Dose of MTX, mg/week	9.4±2.0	10.8±2.9	10.1±2.6	10.1±2.5
Duration of MTX therapy, day (median (IQR))	218.5 (102.5–1241.0)	303.0 (122.0–571.0)	224.0 (112.0–651.0)	194.0 (134.0–546.0)
Prior systemic drug therapies (yes), n (%)	17 (60.7)	15 (51.7)	32 (56.1)	23 (76.7)
Biologicals	8	5	13	11
DMARDs (except MTX)	14	12	26	21
Other	0	0	0	0
No of tender joints, of 68 examined	14.9±12.5	15.9±10.5	15.4±11.4	14.6±5.5
No of swollen joints, of 66 examined	10.6±4.6	12.0±6.3	11.3±5.5	13.6±5.7
Physician global VAS	58.9±23.0	61.4±22.9	60.2±22.8	62.4±18.5
Patient global VAS	52.3±27.1	52.4±27.3	52.4±26.9	53.1±22.0
Patient pain VAS	54.9±27.5	51.3±28.2	53.1±27.7	53.1±22.4
HAQ-DI	0.821±0.673	0.914±0.736	0.868±0.701	0.958±0.611
DAS28-hsCRP	5.0742±0.9281	5.2220±1.1026	5.1494±1.0143	5.1780±0.7796
DAS28-ESR	5.6418±1.0395	5.7726±1.2053	5.7083±1.1188	5.8443±0.8333
hsCRP, mg/dL	1.11±0.95	1.75±1.82	1.43±1.48	1.80±2.20
ESR, mm/hour	35.1±21.9	36.7±18.6	35.9±20.1	43.0±20.3
mTSS	21.345±35.494	21.851±29.122	21.602±32.117	35.178±54.197
Erosion score	8.411±16.407	9.851±15.688	9.143±15.918	15.761±31.925
JSN score	12.935±20.040	12.000±15.405	12.459±17.678	19.417±23.623
Positive RF test at screening, n (%)	25 (89.3)	27 (93.1)	52 (91.2)	30 (100.0)
Positive ACPA test at screening, n (%)	26 (92.9)	28 (96.6)	54 (94.7)	28 (93.3)
IgG, mg/dL	1365.9±295.6	1416.6±314.1	1391.6±303.5	1394.1±291.2
IgM, mg/dL	110.4±50.4	109.6±40.6	110.0±45.3	124.5±54.2
AST, U/L	21.1±6.5	20.6±8.6	20.8±7.6	22.8±12.4
ALT, U/L	22.4±10.5	19.8±14.2	21.1±12.4	21.9±15.8
Leucocytes, 10ˆ9/L	6.69±1.70	7.49±1.66	7.09±1.71	7.30±2.13
Platelets, 10ˆ9/L	310.9±82.4	329.2±99.2	320.2±91.0	371.1±79.5

*All values are mean (±SD) and at baseline, unless otherwise stated.

ACPA, anticitrullinated protein antibodies; ALT, alanine aminotransferase; AST, aspartate aminotransferase; BMI, body mass index; DAS28, 28-joint Disease Activity Score; DMARD, disease-modifying antirheumatic drug; ESR, erythrocyte sedimentation rate; HAQ-DI, Health Assessment Questionnaire-Disability Index; hsCRP, high sensitivity C reactive protein; JSN, joint space narrowing; mTSS, modified Total Sharp Score; MTX, methotrexate; RA, rheumatoid arthritis; RF, rheumatoid factor; VAS, Visual Analogue Scale.

### Primary outcome

In the PPS, patients in the TAS5315 combined group had an ACR20 response rate at week 12 (primary endpoint) of 78.9% (90% confidence interval (CI) 68.1% to 87.4%) compared with 60.0% (90% CI 43.4% to 75.0%) for patients in the placebo group (one-sided p=0.053; table 2, figure 1A). The separate ACR20 response rates were 82.1% and 75.9% with TAS5315 4 mg and 2 mg, respectively.

**Table 2:** Efficacy endpoints at 12 weeks in the per-protocol set

Parameter	TAS5315			Placebo (n=30)
	4 mg (n=28)	2 mg (n=29)	Combined (n=57)	
ACR20 improvement				
Responders, n (%)	23 (82.1)	22 (75.9)	45 (78.9)	18 (60.0)
90% CI	66.1–92.7	59.4–88.1	68.1–87.4	43.4–75.0
P value*	0.058	0.153	0.053	–
ACR50 improvement				
Responders, n (%)	10 (35.7)	9 (31.0)	19 (33.3)	4 (13.3)
95% CI	18.6–55.9	15.3–50.8	21.4–47.1	3.8–30.7
P value*	0.067	0.125	0.072	–
ACR70 improvement				
Responders, n (%)	1 (3.6)	3 (10.3)	4 (7.0)	0 (0.0)
95% CI	0.1–18.3	2.2–27.4	1.9–17.0	0.0–11.6
P value*	0.483	0.112	0.294	–
DAS28-hsCRP				
LS mean	3.3109	3.5024	3.4084	3.9842†, 3.9841‡
LS mean difference§	–0.6733	–0.4818	–0.5757	–
95% CI§	–1.0938, –0.2527	–0.8982, –0.0654	–0.9359, –0.2155	–
hsCRP, mg/dL				
LS mean	0.93	1.11	1.02	1.34
LS mean difference§	–0.42	–0.24	–0.32	–
95% CI§	–0.97, 0.13	–0.78, 0.30	–0.79, 0.14	–
ESR				
LS mean	25.3	31.3	28.3	33.4
LS mean difference§	–8.1	–2.2	–5.1	–
95% CI§	–14.2, –2.1	–8.1, 3.8	–10.3, 0.2	–
SDAI				
LS mean	15.00	13.77	14.37	17.92
LS mean difference§	–2.93	–4.15	–3.55	–
95% CI§	–6.49, 0.64	–7.69, –0.62	–6.60, –0.50	–
CDAI				
LS mean	14.05	12.67	13.35	16.59
LS mean difference§	–2.53	–3.91	–3.24	–
95% CI§	–5.92, 0.85	–7.27, –0.56	–6.14, –0.34	–
HAQ-DI				
LS mean	0.569	0.453	0.510	0.690
LS mean difference§	–0.121	–0.237	–0.181	–
95% CI§	–0.296, 0.053	–0.410, –0.065	–0.331, –0.030	–

*Fisher’s exact test versus placebo.

†For comparison with TAS5315 4 mg and TAS5315 2 mg.

‡For comparison with TAS5315 combined.

§TAS5315 minus placebo.

ACR20, 20% improvement in American College of Rheumatology criteria; CDAI, Clinical Disease Activity Index; DAS28, 28-joint Disease Activity Score; ESR, erythrocyte sedimentation rate; HAQ-DI, Health Assessment Questionnaire-Disability Index; hsCRP, high sensitivity C reactive protein; LS, least squares; SDAI, Simple Disease Activity Index.

### Secondary endpoints

The ACR50 response rate at week 12 was 33.3% in the TAS5315 combined group and 13.3% with placebo (p=0.072; table 2, figure 1B, online supplemental table 3). The ACR70 response rate at week 12 was 7.0% in the TAS5315 combined group and 0.0% in the placebo group (p=0.294; table 2, figure 1C, online supplemental table 3).

**Figure 2:**
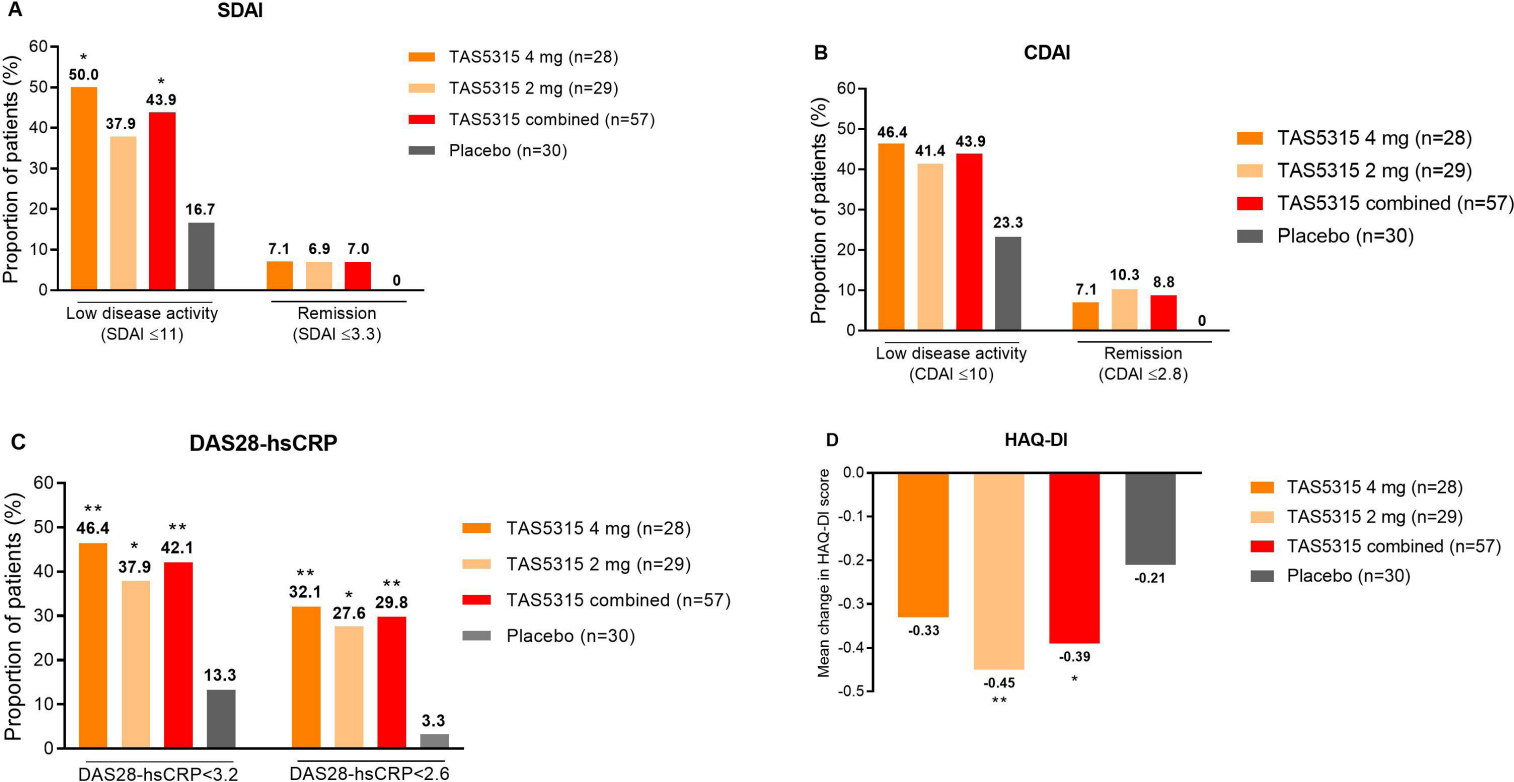
Secondary endpoints at week 12. Proportion of patients meeting (A) Simple Disease Activity Index (SDAI), (B) Clinical Disease Activity Index (CDAI) and (C) Disease Activity Score 28-high sensitivity C reactive protein (DAS28-hSCRP) thresholds for low disease activity or remission, and (D) mean change in Health Assessment Questionnaire-Disability Index (HAQ-DI) score. *p<0.05 vs placebo,**p<0.01 vs placebo.

In the PPS, both Simple Disease Activity Index (SDAI) and Clinical Disease Activity Index (CDAI) scores improved over time up to week 12 in the TAS5315 groups to a greater extent than with placebo (online supplemental table 4). Significantly greater proportions of patients in the TAS5315 combined group met the SDAI criteria for low disease activity (SDAI ≤11) than in the placebo group (figure 2A), and rates of CDAI-defined low disease activity (CDAI≤10) were numerically higher with TAS5315 vs placebo at week 12 (figure 2B).

Similarly, DAS28 based on hsCRP levels (DAS28-hsCRP) decreased (improved) over time up to week 12 in all treatment groups. The mean percentage change from baseline to week 12 was similar in the TAS5315 groups, and was greater than in the placebo group (online supplemental table 4). In addition, a significantly higher proportion of patients achieved DAS28-hsCRP<3.2 or DAS28-hsCRP<2.6 in the TAS5315 groups than in the placebo group at week 12 (figure 2C).

HAQ-DI scores improved over time in all treatment groups, but the magnitude of the change was significantly greater at week 12 in the TAS5315 combined group than the placebo group (p<0.05; table 2, figure 2D).

For ACR20/50/70, SDAI, CDAI, DAS28-hsCRP and HAQ DI, there were numerical differences representing continuing improvement during the full 36 weeks of treatment (online supplemental figure 3).

### Biomarkers

**Figure 3:**
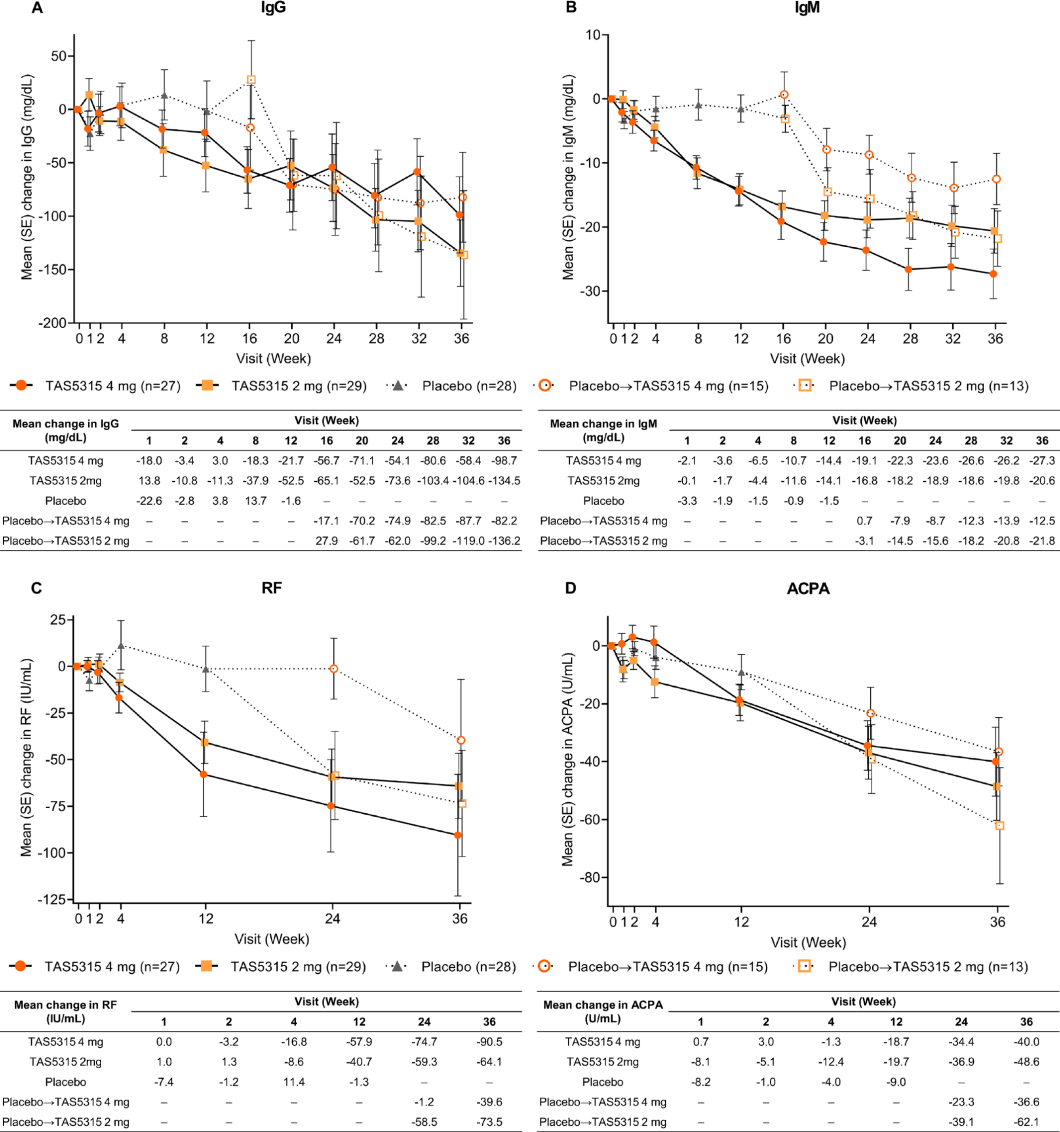
Change over time to week 36 in levels of (A) IgG, (B) IgM, (C) rheumatoid factor (RF) and (D) anticyclic citrullinated protein antibody (ACPA).

The changes in IgG and IgM levels from baseline to week 12 were numerically greater in the TAS5315 groups than the placebo group (figure 3A, B). Similarily, changes in RF and ACPA levels from baseline to week 12 were also numerically greater in the TAS5315 groups than in the placebo group (figure 3C, D).

### Structural joint damage and bone markers

The geometric mean±SD change in mTSS from baseline to week 12 was 1.49±3.75 with TAS5315 4 mg, 2.34±2.46 with TAS5315 2 mg, 1.75±3.19 in the TAS5315 combined group and 1.86±2.58 in the placebo group. The geometric mean±SD change in mTSS from baseline to week 36 was 2.97±4.12 with TAS5315 4 mg, 2.82±2.47 with TAS5315 2 mg, 4.33±1.53 in patients who switched from placebo to TAS5315 4 mg at week 12 and 2.19±4.01 in those who switched to TAS5315 2 mg.

**Figure 4:**
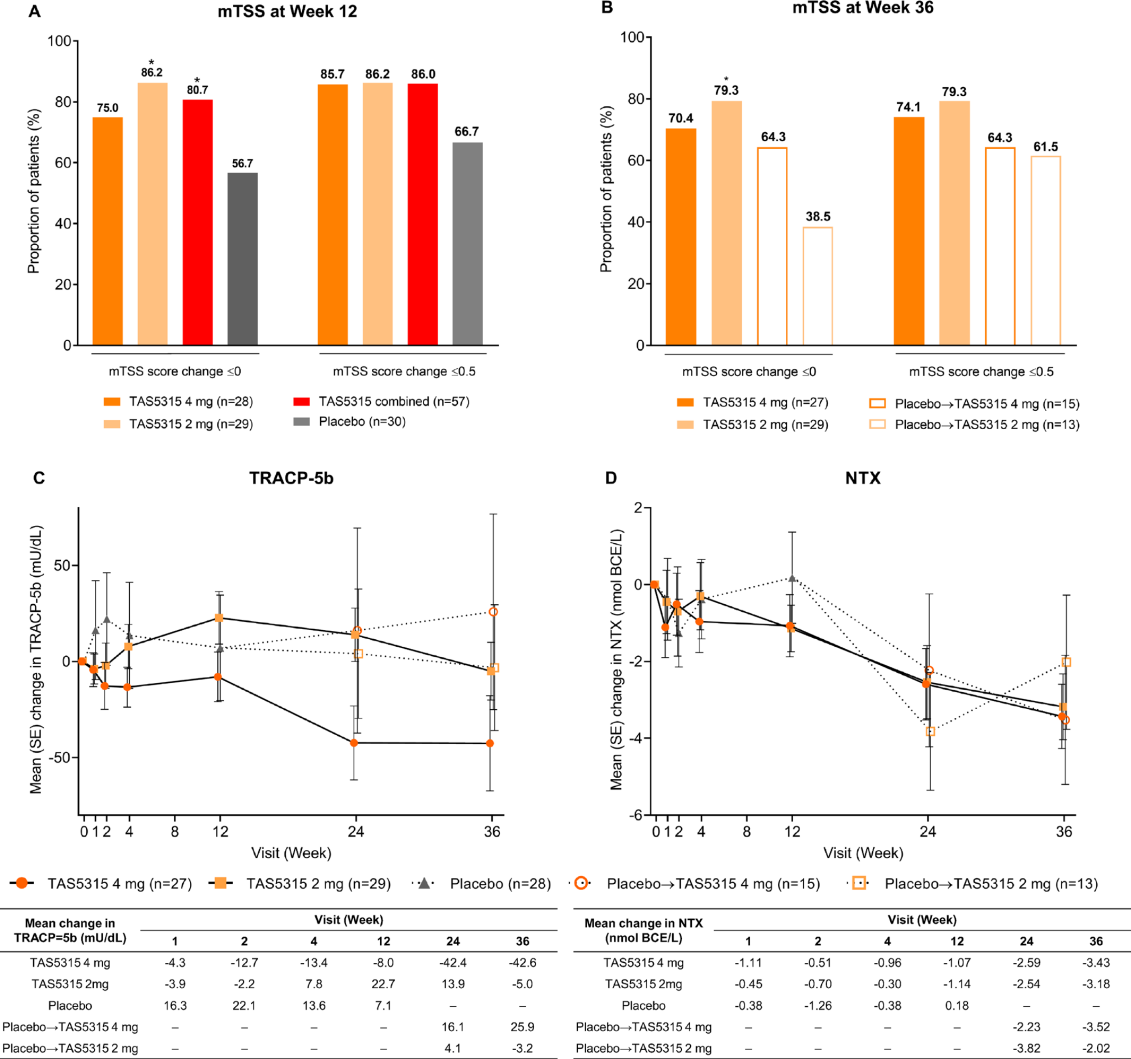
Bone/joint morphology markers. Proportion of patients with modified Total Sharp Score (mTSS) of ≤0 or ≤0.5 at (A) week 12 and (B) week 36. Change over time to week 36 in levels of (C) tartrate-resistant acid phosphatase 5b (TRACP-5b) and (D) N-terminal telopeptide (NTX). *p<0.05 vs placebo (for panel B vs the placebo to TAS5315 4 mg group).

The proportion of patients with no mTSS progression (≤0.5) from baseline to weeks 12 or 36 are shown in figure 4A, B. The number of patients with no mTSS progression tended to be numerically higher in the TAS5315 groups than in the placebo group at week 12 (85.7% with TAS5315 4 mg, 86.2% with TAS5315 2 mg, 86.0% in the TAS5315 combined group and 66.7% with placebo). The cumulative risk of change (increase) from baseline in mTSS tended to be lower in the TAS5313 groups than the placebo group at week 12 (online supplemental figure 4).

In the TAS5315 groups who continued to receive TAS5315 during part B, the proportion of patients with no progression of joint destruction was maintained up to week 36 (74.1% with TAS5315 4 mg and 79.3% with TAS5315 2 mg). Among patients in the placebo group in part A, the proportion with no progression of joint destruction at week 36 after switching to TAS5315 was 64.3% in patients who switched to TAS5315 4 mg and 61.5% in those who switched to TAS5315 2 mg (figure 4B).

There was no significant difference in tartrate-resistant acid phosphatase 5b (TRACP-5b) levels between the TAS5315 groups and the placebo group at any time point, although there were numerical decreases in TRACP-5b levels in part B (from week 12 to week 36) in patients receiving TAS5315 4 mg (figure 4C). N-terminal telopeptide (NTX) showed time-dependent reductions over 36 weeks of treatment (figure 4D).

### Safety

During part A, the incidence of AEs was 41.4% (12/29) with TAS 5315 4 mg, 44.8% (13/29) with TAS5315 2 mg and 57.6% (19/33) with placebo (table 3). All AEs in the TAS5315 groups were of mild-to-moderate severity. The most common AEs in the TAS5315 combined group were nasopharyngitis (10.3%), pruritus (6.9%) and cystitis (5.2%).

**Table 3:** Adverse events (AEs) in all patients who received at least one dose of the study drug (TAS5315 or placebo) in part A

AEs, n (%)	TAS5315			Placebo (n=33)
	4 mg (n=29)	2 mg (n=29)	Combined (n=58)	
Any AE	12 (41.4)	13 (44.8)	25 (43.1)	19 (57.6)
AEs of special interest	1 (3.4)	2 (6.9)	3 (5.2)	1 (3.0)
Any drug-related AE	1 (3.4)	2 (6.9)	3 (5.2)	3 (9.1)
AE by severity category				
Mild AEs	3 (10.3)	4 (13.8)	7 (12.1)	6 (18.2)
Moderate AEs	9 (31.0)	9 (31.0)	18 (31.0)	12 (36.4)
Severe AEs	0	0	0	1 (3.0)
Any serious AE	0	0	0	1 (3.0)
Discontinuation due to AEs	1 (3.4)	0	1 (1.7)	3 (9.1)
AEs leading to death	0	0	0	0
AEs occurring in ≥5% of patients in any group				
Bronchitis	0	1 (3.4)	1 (1.7)	2 (6.1)
Cystitis	1 (3.4)	2 (6.9)	3 (5.2)	0
Nasopharyngitis	3 (10.3)	3 (10.3)	6 (10.3)	4 (12.1)
Pruritus	2 (6.9)	2 (6.9)	4 (6.9)	0

During the 36 weeks of parts A and B, the incidence of AEs was 66.7% (28/42) in the TAS5315 4 mg combined group and 64.3% (27/42) in the TAS5315 2 mg combined group (online supplemental table 5). Only one SAE occurred in the TAS5315 4 mg combined group and was considered to be drug-related. This was a cerebellar haemorrhage that occurred during part B in a patient with concurrent untreated hypertension who received placebo during part A and TAS5315 4 mg in part B. Among patients receiving TAS5315 4 mg, one discontinued treatment during part A because of petechiae and another discontinued during part B because of a limb abscess. Among patients in the placebo group in part A, two discontinued after switching to TAS5315 4 mg during part B because of haemorrhage subcutaneous (n=1) and cerebellar haemorrhage (n=1; described above), and two discontinued after switching to TAS5315 2 mg because of tonsillitis (n=1) and dyspepsia (n=1).

In both parts A and B, 13 AEs of special interest occurred in nine patients during TAS5315 administration. These events were haemorrhage subcutaneous (six events; five with TAS5315 4 mg and one with TAS5315 2 mg), petechiae (three events; two with TAS5315 4 mg and one with TAS5315 2 mg), purpura (two events; one in each TAS5315 dose group), cerebellar haemorrhage (one event with TAS5315 4 mg) and epistaxis (one event with TAS5315 2 mg).

Over 36 weeks, nine patients experienced bleeding events, of whom four and two patients recovered with drug continuation and interruption, respectively. Three patients recovered with discontinuation of TAS5315. Except for a single severe cerebellar haemorrhage event, all other AEs of special interest were of mild severity.

Overall, there was no significant difference in AE incidence between the TAS5315 combined group and the placebo group in part A (12 weeks) of this study, although the likelihood of TAS5315 causing bleeding-related AEs cannot be denied. No dose-dependent increases in AEs were observed with TAS5315. There were no clinically significant changes or abnormalities in laboratory data, vital signs or 12-lead electrocardiography.

## Discussion

In this phase II study of TAS5315 in Japanese patients with RA, the ACR20 response rate at week 12 was numerically higher in the TAS5315 combined group than in the placebo group, but this difference was not statistically significant and the study did not meet the primary endpoint. ACR 50/70 improvement rates and other endpoints reflecting the disease activity of RA, such as SDAI, CDAI and DAS28-hsCRP, also showed some numerical differences between TAS5315 and placebo. Despite the study not achieving its primary endpoint, the numerical differences noted between TAS5315 and placebo may be worth pursing in terms of mechanism.

In the current study, following the completion of part A, patients who showed an improvement of ≥20% in the number of painful (tender) or swollen joints compared with baseline continued to part B. In part B, all patients were administered TAS5315 treatment. At the end of part A, an unexpectedly high ACR20 response rate was observed in the placebo group. A subanalysis of Japanese individuals with RA who participated in the RA-BEAM trial reported a placebo ACR20 response rate at week 12 of 34%,^21^ whereas in the current study, the ACR20 response rate with placebo was 60%. The ACR20 response rate in the TAS5315 combined group was also higher than previously noted with other biologic agents for example, etanercept, tocilizumab and adalimumab.^3^ These discrepancies may be attributed to poor site selection and study design. The effect of bias is likely to be accentuated due to the large number of clinics that were selected in the study, resulting in a higer ACR20 response rate in the placebo group. Additionally, it is possible that physicians’ evaluations of painful (tender) or swollen joints were biased and that the use of non-specific RA classification criteria for the continuation of patients to part B potentially resulted in study investigators including individuals who did not have active RA disease. Furthermore, since ACR20 includes both objective and subjective (VAS) measures of disease activity, patient expectations might have influenced both patient and physician assessments.^22^ Patients with RA refractory to MTX have been reported to have had high ACR20 responses when previously administered MTX was switched to sponsor-providing blinded MTX in the monotherapy trial of upadacitinib.^23^ Clinical responses to placebo have been reported to be higher in patients who had continued an insufficient MTX background therapy.^24^ Similar effects may have occurred in some patients with high ACR20 response rates due to having received the study treatment in the current study. A meta-analysis of randomised controlled trials of biologicals or JAK inhibitors in RA also reported increased placebo response rates for ACR20, ACR50 and ACR70 over the last 20 years, which were statistically significant in the case of the more sensitive measures (ie, ACR50 and ACR70).^22^ The observation that DAS28(CRP) responses were also numerically better than most other endpoints may also be partly explained by study design and physician assessment bias.

Biomarkers, such as autoantibodies (RF, ACPA) and IgG, IgM, showed a time-dependent decrease up to week 36 in the TAS5315 groups. Data from an animal model of RA indicated that TAS5315 suppresses proliferation and activation of antibody-producing plasma cells,^16 17 25 26^ and biomarker data from the current study support a similar mechanism in patients with RA.

mTSS, which is used to evaluate the progression of joint destruction, was lower in the TAS5315 groups than in the placebo group at week 12, and approximately 19% more patients in the TAS5315 combined group than in the placebo group showed no progression at week 12 (using a change from baseline in mTSS of ≤0.5).^27^ This suggests that TAS5315 might have an inhibitory effect on the progression of joint destruction. TRACP-5b is a bone resorption marker which is a direct index of osteoclast number and bone resorption activity.^28 29^ TRACP-5b levels with TAS5315 4 mg numerically decreased from week 12 to week 36, although the levels were not significantly different at any time point between the TAS5315 and placebo groups. This signal of a potential effect requires further analysis.

Numerically lower levels of NTX, a decomposition product of bone collagen, were observed in the TAS5315 groups than in the placebo group at week 12. NTX levels in the TAS5315 groups also showed a time-dependent decrease up to week 36. These changes reflect the mechanism of action of TAS5315 on BTK inhibition, since BTK is involved in osteoclast maturation via receptor activation of nuclear factor kappa B ligand, as well as osteoclast differentiation.^30^

In part A, the incidence of AEs was not significantly different between the TAS5315 and placebo groups; there were no dose-dependent increases in AEs with TAS5315 4 mg or 2 mg throughout the study. Moreover, despite a decrease in IgG and IgM levels over time in the TAS5315 groups, there was no evidence that this was associated with an increased risk of infection.

Throughout the study, three patients discontinued TAS5315 (one in part A and two in part B) because of bleeding-related AEs, specifically petechiae (n=1), subcutaneous haemorrhage (n=1) and cerebellar haemorrhage (n=1). The only SAE was cerebellar haemorrhage. All of these bleeding-related AEs resolved with continued TAS5315 administration, or after discontinuation or interruption of the study drug. Overall, TAS5315 may have acceptable tolerability, but based on individual events and the pharmacological mechanism of TAS5315, we cannot rule out the possibility that TAS5315 may cause bleeding-related AEs. It should also be noted that the exclusion criteria (eg, history of haemorrhagic diseases) were established considering the bleeding risks associated with TAS5315, which may have reduced the bleeding rate observed in the current study.

Notably, the completion rate of patients who switched from placebo to TAS5315 was numerically lower than those who received TAS5315 for 36 weeks (ie, in part A and B). A potential reason for this difference may be disease progression in patients receiving placebo during part A.

Ibrutinib, an irreversible BTK inhibitor used for the treatment of B-cell malignancies, has been reported to cause bleeding, for which one of the causes is suppression of collagen-induced platelet aggregation.^31^ A phase I study in healthy adults found suppression of platelet aggregation during TAS5315 treatment.^19^ The mechanism appears to be the same as ibrutinib-induced suppression of platelet aggregation, since the effect is limited to platelet aggregation stimulated by collagen, with adenosine diphosphate-induced platelet aggregation spared.

Historically, there have been some ineffective BTK inhibitors as therapeutic agents for RA (eg, spebrutinib and poseltinib), with fenebrutinib being one of the few successful agents for RA treatment with this mechanism of action.^32^ Despite the current study failing to show significant changes in the ACR20 response rates, changes in IgG, IgM, RF and ACPA biomarkers were similar between TAS5315 and those previously reported with fenebrutinib.^32^ Although fenebrutinib binds to BTK in a different way to TAS5315, its mechanism of action is largely similar. Conducting a new trial of TAS5315 adjusting dose and treatment timings (eg, inadequate response to JAK inhibitor) and disease duration, as well as appropriate study site selections and accurate enrolment of patients with diagnosed RA might generate more meaningful and insightful efficacy results.

There are several limitations in this study. The relatively small sample size limits the generalisability of these results. Further, because only patients who achieved a 20% improvement in pain and SJC at week 12 were able to transition to the subsequent 24-week treatment period, selection bias (in favour of responding patients) may have affected results at week 36. In addition, the efficacy of TAS5315 against joint destruction was only assessed up to week 36; longer-term studies are needed to evaluate the effects of TAS5315 on joint changes.

In conclusion, the primary endpoint was not achieved in this phase II study in Japanese patients with RA. TAS5315 appeared to have some bleeding risks but nevertheless, demonstrated numerical differences compared with placebo in the improvement rates of all measures of RA disease activity. Future analysis of the risk–benefit of TAS5315 for RA should be considered.

## Data Availability

Data are available on reasonable request. The sponsor policy on data sharing may be found at https://www.taiho.co.jp/en/science/policy/clinical_trial_information_disclosure_policy/index.html.
