# Supplementary material for: Irreversible covalent Bruton’s tyrosine kinase inhibitor, TAS5315 versus placebo in rheumatoid arthritis patients with inadequate response to methotrexate: a randomised, double-blind, phase IIa trial
Source: Ann Rheum Dis. 2023 May 22;82(8):1025–34. doi: 10.1136/ard-2022-223759 (PMC10359550; doi:10.1136/ard-2022-223759)
Supplement: Supplementary data [file ard-2022-223759supp001.pdf]

SUPPLEMENTAL MATERIAL

Study investigators

Akira Sagawa, Yujiro Kon, Fuminori Hirano, Osamu Takai, Takanori Azuma, Shuji Ohno, Wataru Hirose, Ryutaro Matsumura, Akiko Mitsuo, Toshiyuki Kaneko, Naooki Katsuyama, Masanao Asano, Norihiko Koido, Kazunori Sugimoto, Rei Ito, Kenji Kondo, Shigenori Tamaki, Motokazu Kai, Hideo Hashimoto, Akira Okamoto, Michinori Ishitoku, Keisuke Oda, Hajime Miyazato, Takeshi Tokito, Yohei Takeuchi, Takahito Kimata, Eisuke Shono, Masahiko Tsuboi, Taiichiro Miyashita, Yukitaka Ueki, Kenmei Sakata, Tadashi Nakamura, Motohiro Oribe, Yasuo Suenaga, Takashi Ohira, Mutsuaki Kai, Yasushi Tanaka, Yuji Yamanishi, Tsukasa Matsubara, Yoichiro Haji, Koji Kuroda, Kou Katayama, Kazuhide Tanimura, Seiji Tsuboi, Shigeo Irimajiri, Shuzo Yoshida, Shinichi Ishioka

Supplemental methods

Randomisation of patients on placebo for Part B

Patients who met three transition criteria at the 12-week treatment visit (i.e. at the end of Part A) were enrolled and randomised in Part B.

Transition criteria were as follows:

1. Improvement of ≥20% in the number of painful (tender) joints or swollen joints at the 12-week visit of the treatment period compared with baseline.
2. No safety issues/severe side effects observed by the investigator or sub-investigator at the 12th week of treatment.
3. The investigator or sub-investigator has judged that the observation/test items

specified in Part B can be performed and the drug can be continued appropriately.

Indicators of structural joint damage

Structural joint damage due to rheumatoid arthritis (RA) was assessed from plain X-ray images of the hands and feet using the van der Heijde modification of the total Sharp score[1] (mTSS; range 0 to 448) and its composite scores, including the bone erosion score and the joint space narrowing (JSN) score. Bone erosion and JSN scores were determined by central review of the images performed at the radiology review centre, with the average score between two readers used for this analysis.

Statistical analysis

Target enrolment was 90 patients (30 per treatment group in Part A) to account for dropouts. The full analysis set (FAS) included all treated patients in Part A who were evaluated for at least one primary or secondary efficacy endpoint after the initiation of study drug administration. The per-protocol set (PPS) included all patients in the FAS who continued to meet study eligibility criteria, were ≥80% compliant with treatment in part A and did not have any protocol violations.

Demographic and clinical characteristics were summarised using descriptive statistics. The primary endpoint was assessed in the PPS using non-responder imputation (NRI) to account for missing data, and the between-group comparison of the TAS5315 4 and 2 mg combined group (TAS5315 combined group) and the placebo group was undertaken using Fisher's exact test. Two analyses were performed for the sensitivity analysis assessment of the primary endpoint. The first analysis was similar to the main primary endpoint analysis (except the full analysis set [FAS] rather than the per-protocol set [PPS] was used). The second analysis was also similar to

the main primary endpoint analysis but used 'last observation carried forward' (LOCF) as the imputation method.

The rate of categorical secondary endpoints in treatment versus placebo groups was compared using Fisher's exact test, with missing data imputed by NRI and/or last observation carried forward (LOCF). Continuous variables were analysed by the analysis of covariance using the baseline values as covariates. The least-squares means (LSMs), the differences in the LSMs with the placebo group at each time point, and the 95% confidence intervals for the differences were calculated. Estimates of the individual measures in the ACR core set, Disease Activity Score 28 (DAS28)-high sensitivity C-reactive protein (hsCRP), DAS28-erythrocyte sedimentation rate (ESR), Clinical Disease Activity Index (CDAI) and Simple Disease Activity Index (SDAI) were calculated using the mixed-effect models for repeated measures (MMRM).

For mTSS, the geometric mean ± standard deviation values were calculated using non-zero records, and the cumulative probability plot of the change in the measured values from baseline was prepared. Missing data were imputed, using the linear extrapolation method. A geometric mean rather than the absolute change was reported as mTSS values are exponential in nature.

Statistical analyses were performed using SAS statistical software (version 9.4; SAS Institute Inc, Cary, North Carolina).

Supplemental Table 1 Key exclusion criteria

**Exclusion criterion**
Prior use of any JAK inhibitor or BTK inhibitor
Recent use of other treatments for RA (etanercept, interferon or conventional DMARDs other than MTX within the past 28 days; injected corticosteroids [intramuscular, intravenous or intra-articular], intra-articular hyaluronic acid or physical therapy within the past 14 days; infliximab or ticilizumab within 56 days; leflunomide within 90 days; rituximab within 1 year)
A history of or current systemic inflammatory or autoimmune diseases other than RA, active fibromyalgia, or a diagnosis of Felty's syndrome
Use of treatments for osteoporosis (e.g. denosumab or zoledronic acid) at intervals of >1 month within 2 years
Active or chronic HIV or hepatitis B or C infections
History of surgical treatment (e.g. synovectomy or arthroplasty)
History of drug hypersensitivity or haemorrhagic diseases (such as thrombocytopenic purpura, bleeding tendency caused by vascular disorders, haemophilia, and other blood coagulation disorders)

BTK, Bruton's tyrosine kinase; DMARDs, disease-modifying antirheumatic drugs; HIV, human immunodeficiency virus; JAK, Janus kinase; MTX, methotrexate; RA, rheumatoid arthritis.

Takeuchi T, *et al. Ann Rheum Dis* 2023; 82:1025–1034. doi: 10.1136/ard-2022-223759

Supplemental Table 2 Secondary endpoints

Endpoint	Assessment time point
ACR20	Week 36
ACR50	Weeks 12 and 36
ACR70	Weeks 12 and 36
ACR core set[2]	Weeks 12 and 36
SDAI	Weeks 12 and 36
CDAI	Weeks 12 and 36
HAQ-DI	Weeks 12 and 36
DAS28-hsCRP	Weeks 12 and 36
DAS28-ESR	Weeks 12 and 36
hsCRP	Weeks 12 and 36
ESR	Weeks 12 and 36
RF test	Weeks 12 and 36
ACPA test	Weeks 12 and 36
IgG	Weeks 12 and 36
IgM	Weeks 12 and 36
mTSS	Weeks 12 and 36
mTSS, configuration score	Weeks 12 and 36
TRACP-5b level	Weeks 12 and 36
NTX level	Weeks 12 and 36

ACPA, anti-cyclic citrullinated peptide antibody; ACR20, American College of Rheumatology 20% response; ACR50, American College of Rheumatology 50% response; ACR70, American College of Rheumatology 70% response; CDAI, Clinical Disease Activity Index; DAS28, Disease Activity Score-28; ESR, erythrocyte sedimentation rate; Ig, immunoglobulin G; IgM, immunoglobulin M; mTSS, modified total Sharp score; NTX, N-terminal telopeptide; RF, rheumatoid factor; SDAI, Simple Disease Activity Index; TRACP-5b, tartrate-resistance acid phosphatase.

Supplemental Table 3 Response rates at Weeks 2, 4, 8 and 12 in the per-protocol set

**Parameter**	**Response rate, n (%) [95% CI]**			
	**TAS5315 4 mg (n=28)**	**TAS5315 2 mg (n=29)**	**TAS5315 combined (n=57)**	**Placebo (n=30)**
**ACR20 improvement**				
Week 2	6 (21.4) [8.3–41.0]	8 (27.6) [12.7–47.2]	14 (24.6) [14.1–37.8]	3 (10.0) [2.1–26.5]
p-value[a]	0.290	0.104	0.155	–
Week 4	15 (53.6) [33.9–72.5]	8 (27.6) [12.7–47.2]	23 (40.4) [27.6–54.2]	5 (16.7) [5.6–34.7]
p-value[a]	**0.005**	0.360	**0.030**	–
Week 8	21 (75.0) [55.9–89.3]	15 (51.7) [32.5–70.6]	36 (63.2) [49.3–75.6]	11 (36.7) [19.9–56.1]
p-value[a]	**0.004**	0.299	**0.024**	–
Week 12	23 (82.1) [63.1–93.9]	22 (75.9) [56.5–89.7]	45 (78.9) [66.1–88.6]	18 (60.0) [40.6–77.3]
p-value[a]	0.086	0.267	0.079	–
**ACR50 improvement**				
Week 2	1 (3.6) [0.1–18.3]	2 (6.9) [0.8–22.8]	3 (5.3) [1.1–14.6]	0 (0.0) [0.0–11.6]
p-value[a]	0.483	0.237	0.548	–
Week 4	4 (14.3) [4.0–32.7]	4 (13.8) [3.9–31.7]	8 (14.0) [6.3–25.8]	0 (0.0) [0.0–11.6]
p-value[a]	**0.048**	0.052	**0.047**	–
Week 8	6 (21.4) [8.3–41.0]	4 (13.8) [3.9–31.7]	10 (17.5) [8.7–29.9]	3 (10.0) [2.1–26.5]
p-value[a]	0.290	0.706	0.529	–
Week 12	10 (35.7) [18.6–55.9]	9 (31.0) [15.3–50.8]	19 (33.3) [21.4–47.1]	4 (13.3) [3.8–30.7]
p-value[a]	0.067	0.125	0.072	–
**ACR70 improvement**				
Week 2[a]	0 (0.0) [0.0–12.3]	0 (0.0) [0.0–11.9]	0 (0.0) [0.0–6.3]	0 (0.0) [0.0–11.6]
p-value[a]	–	–	–	–
Week 4	0 (0.0) [0.0–12.3]	2 (6.9) [0.8–22.8]	2 (2.3) [0.4–12.1]	0 (0.0) [0.0–11.6]
p-value[a]	–	0.237	0.543	–
Week 8	2 (7.1) [0.9–23.5]	2 (6.9) [0.8–22.8]	4 (7.0) [1.9–17.0]	0 (0.0) [0.0–11.6]

p-value[a]	0.229	0.237	0.294	–
Week 12	1 (3.6) [0.1–18.3]	3 (10.3) [2.2–27.4]	4 (7.0) [1.9–17.0]	0 (0.0) [0.0–11.6]
p-value[a]	0.483	0.112	0.294	–

[a]Fisher's exact test versus placebo. Significant values are shown in bold.

ACR20, 20% improvement in American College of Rheumatology criteria; ACR50, 50% improvement in American College of Rheumatology criteria; ACR70, 70% improvement in American College of Rheumatology criteria; CI, confidence interval.

Supplemental Table 4 Mean percentage change from baseline in SDAI, CDAI and DAS28-hsCRP at Week 12 in the per-protocol set

Parameter	Mean ± SD percentage change from baseline (%)			
	**TAS5315 4 mg**	**TAS5315 2 mg**	**TAS5315 combined**	**Placebo**
SDAI	–57.1 ± 22.7	–57.9 ± 20.0	–57.5 ± 21.2	–44.1 ± 24.3
CDAI	–57.6 ± 23.4	–59.3 ± 21.0	–58.5 ± 22.0	–46.1 ± 23.2
DAS28-hsCRP	–36.6 ± 17.6	–32.3 ± 14.3	–34.4 ± 16.0	–22.2 ± 16.4

CDAI, Clinical Disease Activity Index; DAS28-hsCRP, 28-joint Disease Activity Score based on high-sensitivity C-reactive protein levels; SD, standard deviation; SDAI, Simple Disease Activity Index.

Supplemental Table 5 Adverse events during Part A and Part B

AEs, n (%)	TAS5315 4 mg (n=42)[a]	TAS5315 2 mg (n=42)[b]
Any AE	28 (66.7)	27 (64.3)
AEs of special interest[c]	4 (9.5)	4 (9.5)
Any drug-related AE	7 (16.7)	7 (16.7)
AE by severity category		
Mild AEs	6 (14.3)	3 (7.1)
Moderate AEs	21 (50.0)	24 (57.1)
Severe AEs	1 (2.4)	0
Any serious AE	1 (2.4)	0
Discontinuation due to AEs	3 (7.1)	2 (4.8)
AEs leading to Death	0	0
AEs occurring in ≥2 patients in any group		
Hepatic function abnormal	4 (9.5)	0
Nasopharyngitis	7 (16.7)	8 (19.0)
Gastroenteritis	3 (7.1)	0
Cystitis	1 (2.4)	2 (4.8)
Pharyngitis	0	2 (4.8)
Tonsillitis	0	2 (4.8)
Contusion	2 (4.8)	1 (2.4)
ALT increased	1 (2.4)	2 (4.8)
Back pain	2 (4.8)	2 (4.8)
Haemorrhage subcutaneous	2 (4.8)	1 (2.4)
Oropharyngeal pain	2 (4.8)	1 (2.4)
Pruritus	1 (2.4)	3 (7.1)
Urticaria	2 (4.8)	1 (2.4)

[a]Includes patients who received TAS5315 4 mg in Parts A and B, and those who received placebo in Part A and TAS5315 4 mg in Part B.

[b]Includes patients who received TAS5315 2 mg in Parts A and B, and those who received placebo in Part A and TAS5315 2 mg in Part B.

[c]Nine patients experienced bleeding events across Parts A and B of the study. Of these patients, one patient discontinued treatment in Part A (mild bleeding event) and has been excluded from this table.

AE, adverse event; ALT, alanine aminotransferase.

Takeuchi T, *et al. Ann Rheum Dis* 2023; 82:1025–1034. doi: 10.1136/ard-2022-223759

Supplemental Figure 1 Study design. MTX, methotrexate

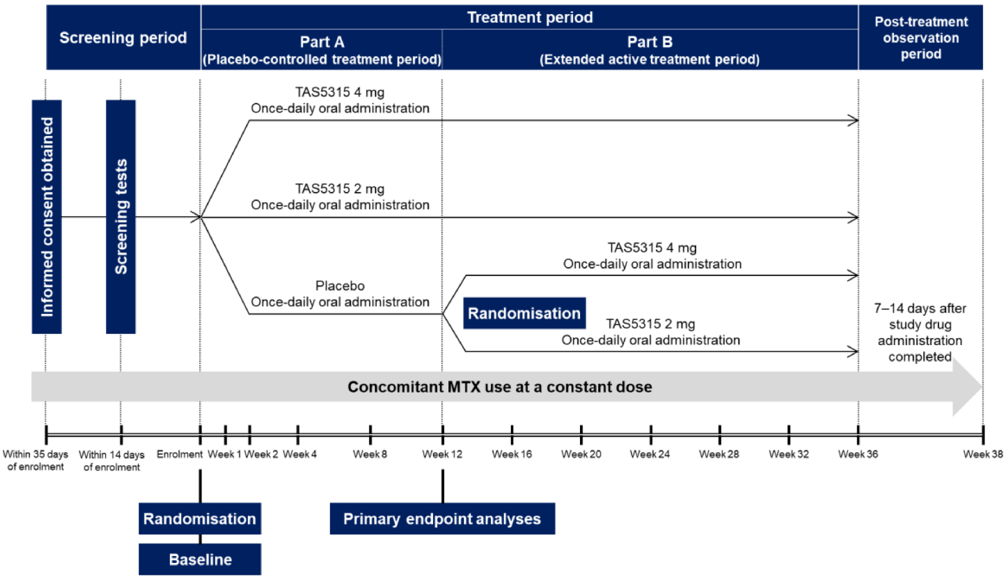

Supplemental Figure 2 Patient disposition. AE, adverse event; SAE, serious adverse event; SJC, swollen joint count; TJC, tender joint count.

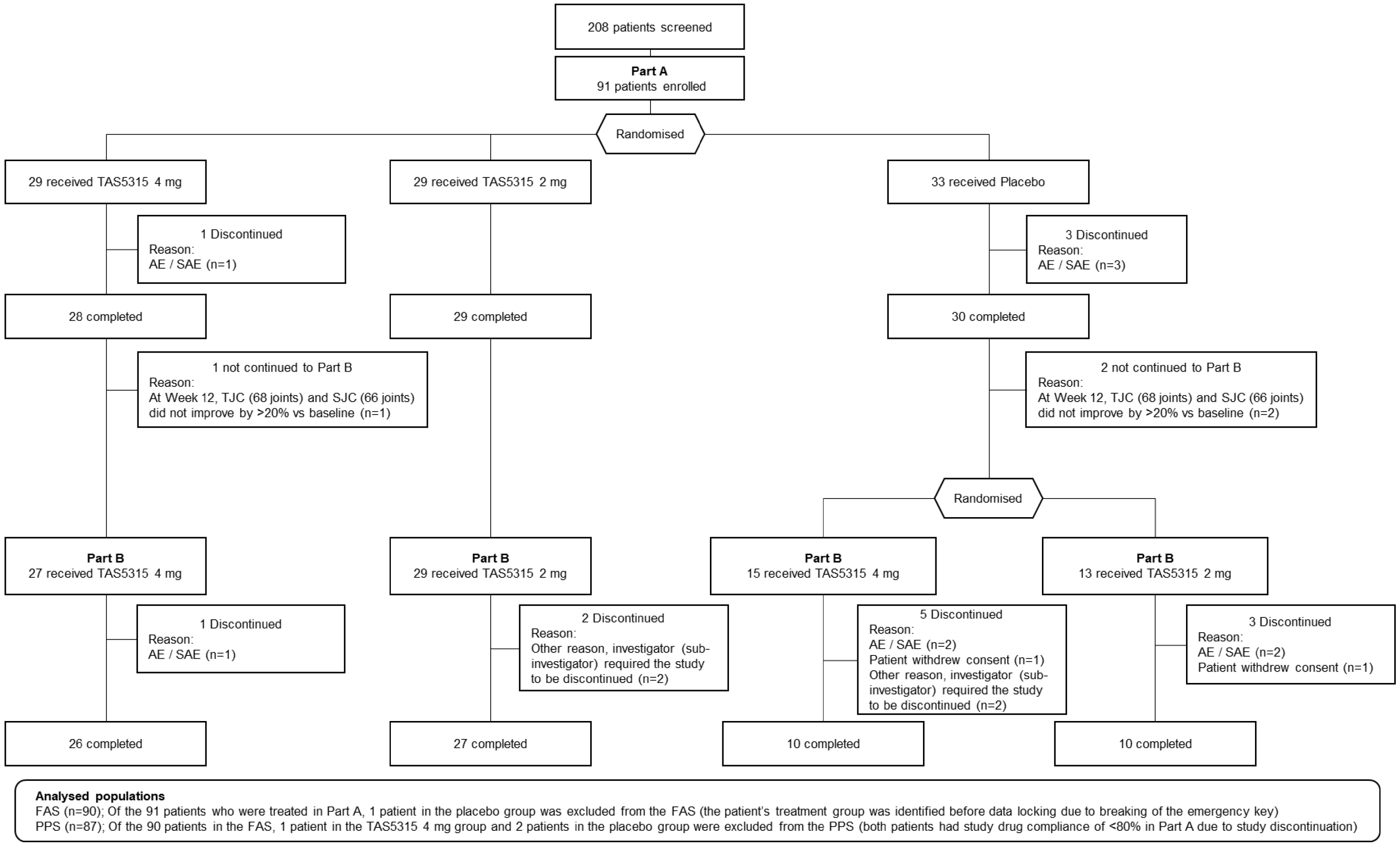

BMJ Publishing Group Limited (BMJ) disclaims all liability and responsibility arising from any reliance
placed on this supplemental material which has been supplied by the author(s)

Supplemental Figure 3 Change over time to Week 36 in the proportion of patients with (A) 20% improvement in American College of Rheumatology (ACR) criteria (ACR20), (B) ACR50 and (C) ACR70, and the change from baseline in (D) Simple Disease Activity Index (SDAI), (E) Clinical Disease Activity Index (CDAI), (E) 28-joint Disease Activity Score based on high sensitivity C-reactive protein levels (DAS28-hsCRP), and (G) Health Assessment Questionnaire-Disability Index (HAQ-DI). SE, standard error.

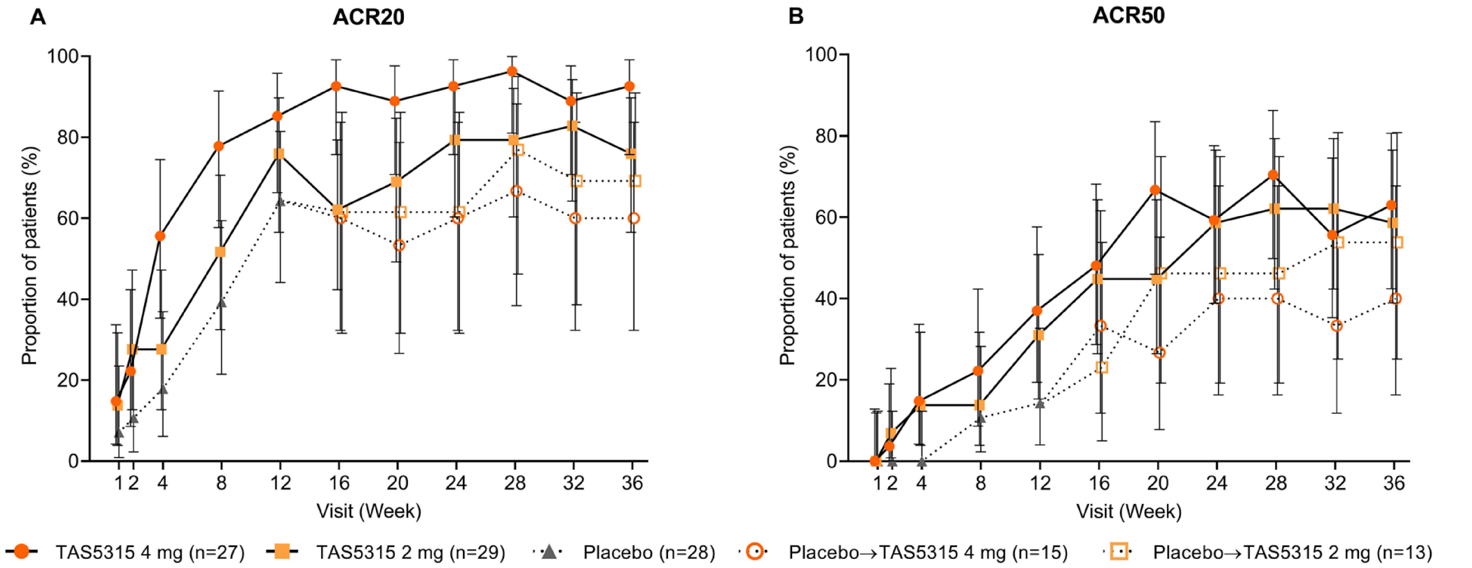

Proportion of patients with ACR20 (%)	Visit (Week)										
	1	2	4	8	12	16	20	24	28	32	36
TAS5315 4 mg	14.8	22.2	55.6	77.8	85.2	92.6	88.9	92.6	96.3	88.9	92.6
TAS5315 2mg	13.8	27.6	27.6	51.7	75.9	62.1	69.0	79.3	79.3	82.8	75.9
Placebo	7.1	10.7	17.9	39.3	64.3	–	–	–	–	–	–
Placebo→TAS5315 4 mg	–	–	–	–	–	60.0	53.3	60.0	66.7	60.0	60.0
Placebo→TAS5315 2 mg	–	–	–	–	–	61.5	61.5	61.5	76.9	69.2	69.2

Proportion of patients with ACR50 (%)	Visit (Week)										
	1	2	4	8	12	16	20	24	28	32	36
TAS5315 4 mg	0.0	3.7	14.8	22.2	37.0	48.1	66.7	59.3	70.4	55.6	63.0
TAS5315 2mg	0.0	6.9	13.8	13.8	31.0	44.8	44.8	58.6	62.1	62.1	58.6
Placebo	0.0	0.0	0.0	10.7	14.3	–	–	–	–	–	–
Placebo→TAS5315 4 mg	–	–	–	–	–	33.3	26.7	40.0	40.0	33.3	40.0
Placebo→TAS5315 2 mg	–	–	–	–	–	23.1	46.2	46.2	46.2	53.8	53.8

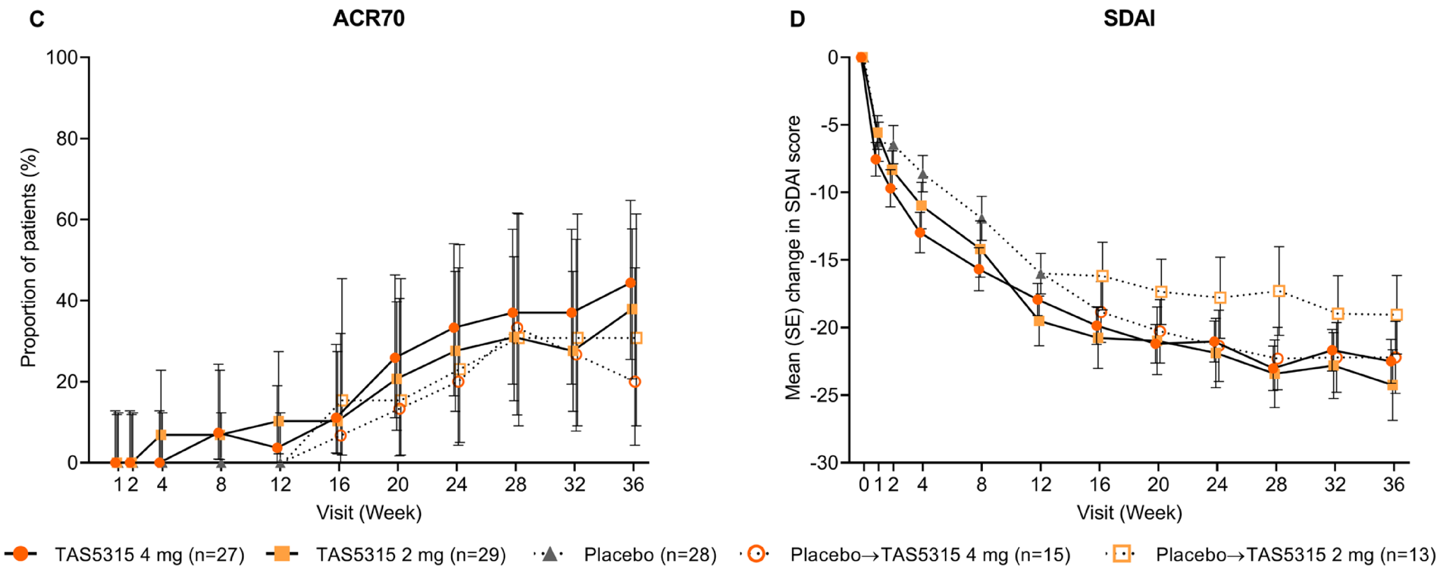

Proportion of patients with ACR70 (%)	Visit (Week)										
	1	2	4	8	12	16	20	24	28	32	36
TAS5315 4 mg	0.0	0.0	0.0	7.4	3.7	11.1	25.9	33.3	37.0	37.0	44.4
TAS5315 2mg	0.0	0.0	6.9	6.9	10.3	10.3	20.7	27.6	31.0	27.6	37.9
Placebo	0.0	0.0	0.0	0.0	0.0	–	–	–	–	–	–
Placebo→TAS5315 4 mg	–	–	–	–	–	6.7	13.3	20.0	33.3	26.7	20.0
Placebo→TAS5315 2 mg	–	–	–	–	–	15.4	15.4	23.1	30.8	30.8	30.8

Mean change in SDAI score	Visit (Week)										
	1	2	4	8	12	16	20	24	28	32	36
TAS5315 4 mg	-7.6	-9.7	-13.0	-15.7	-17.9	-19.9	-21.2	-21.0	-23.0	-21.7	-22.5
TAS5315 2mg	-5.6	-8.3	-11.0	-14.2	-19.5	-20.8	-21.0	-21.9	-23.4	-22.8	-24.3
Placebo	-6.2	-6.5	-8.6	-11.9	-16.0	–	–	–	–	–	–
Placebo→TAS5315 4 mg	–	–	–	–	–	-18.8	-20.3	-21.4	-22.3	-22.2	-22.2
Placebo→TAS5315 2 mg	–	–	–	–	–	-16.2	-17.4	-17.8	-17.3	-19.0	-19.1

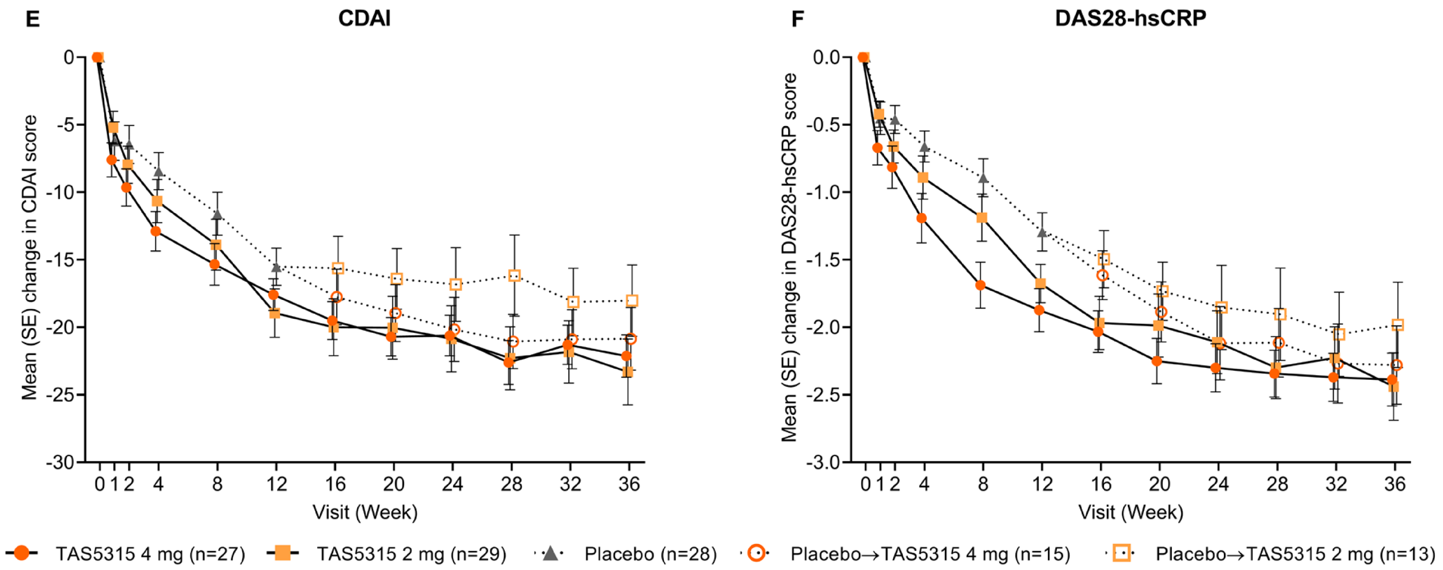

Mean change in CDAI score	Visit (Week)										
	1	2	4	8	12	16	20	24	28	32	36
TAS5315 4 mg	-7.6	-9.6	-12.9	-15.3	-17.6	-19.5	-20.7	-20.6	-22.6	-21.3	-22.1
TAS5315 2mg	-5.2	-8.0	-10.7	-13.9	-19.0	-20.0	-20.0	-20.9	-22.3	-21.8	-23.3
Placebo	-6.2	-6.5	-8.4	-11.6	-15.5	–	–	–	–	–	–
Placebo→TAS5315 4 mg	–	–	–	–	–	-17.8	-19.0	-20.2	-21.1	-20.9	-20.9
Placebo→TAS5315 2 mg	–	–	–	–	–	-15.6	-16.4	-16.8	-16.2	-18.1	-18.0

Mean change in DAS28-hsCRP score	Visit (Week)										
	1	2	4	8	12	16	20	24	28	32	36
TAS5315 4 mg	-0.67	-0.81	-1.19	-1.69	-1.87	-2.03	-2.25	-2.30	-2.34	-2.37	-2.39
TAS5315 2mg	-0.42	-0.66	-0.89	-1.19	-1.68	-1.97	-1.99	-2.11	-2.30	-2.23	-2.44
Placebo	-0.45	-0.46	-0.66	-0.89	-1.29	–	–	–	–	–	–
Placebo→TAS5315 4 mg	–	–	–	–	–	-1.61	-1.89	-2.12	-2.11	-2.27	-2.28
Placebo→TAS5315 2 mg	–	–	–	–	–	-1.50	-1.73	-1.85	-1.90	-2.05	-1.98

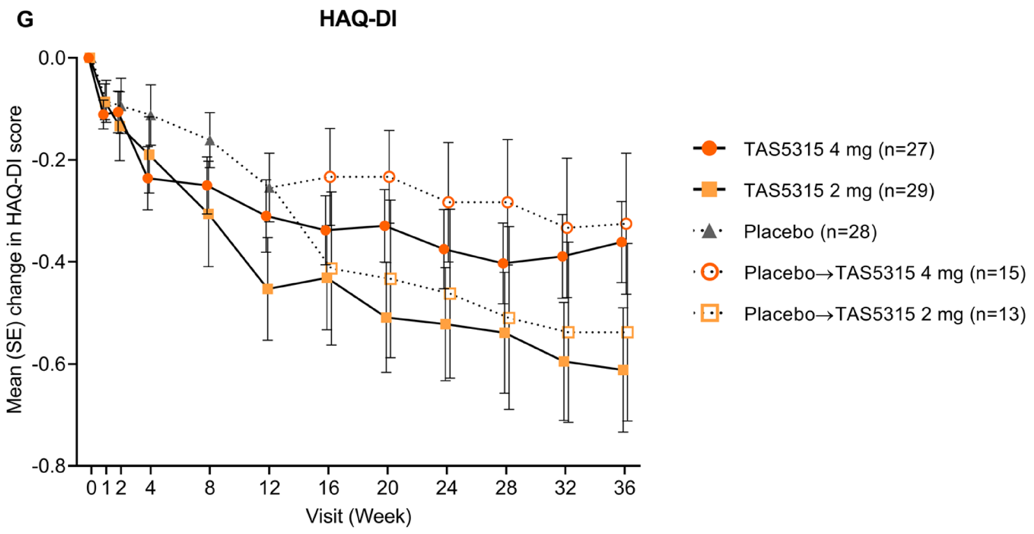

Mean change in HAQ-DI score	Visit (Week)										
	1	2	4	8	12	16	20	24	28	32	36
TAS5315 4 mg	-0.11	-0.11	-0.24	-0.25	-0.31	-0.34	-0.33	-0.38	-0.40	-0.40	-0.36
TAS5315 2mg	-0.09	-0.13	-0.19	-0.31	-0.45	-0.43	-0.51	-0.52	-0.54	-0.60	-0.61
Placebo	-0.09	-0.09	-0.11	-0.16	-0.25	–	–	–	–	–	–
Placebo→TAS5315 4 mg	–	–	–	–	–	-0.23	-0.23	-0.28	-0.28	-0.33	-0.33
Placebo→TAS5315 2 mg	–	–	–	–	–	-0.41	-0.43	-0.46	-0.51	-0.54	-0.54

Supplemental Figure 4 Cumulative probability plot of change in modified total Sharp score (mTSS) from baseline at (A) Week 12 and (B) Week 36

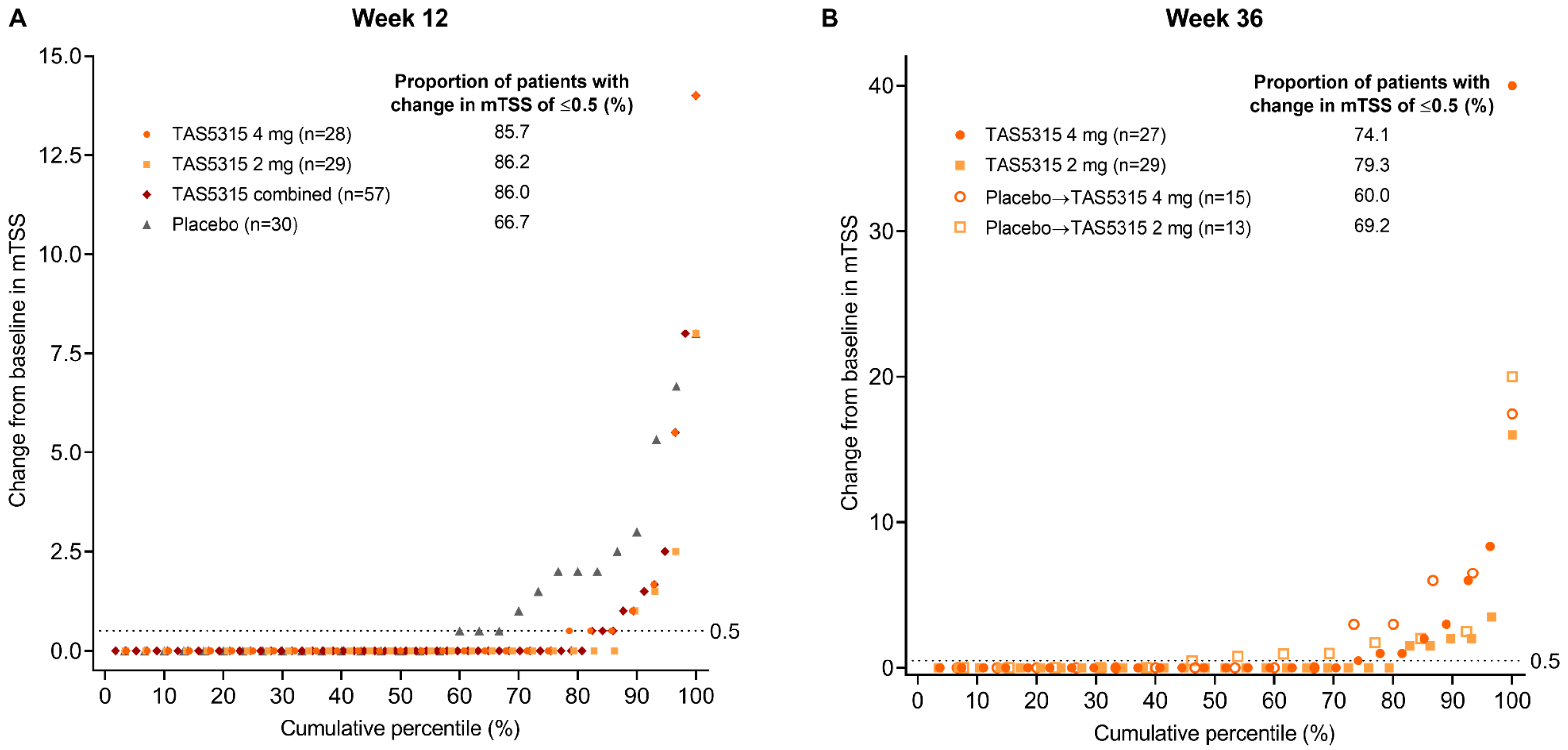

References

1 van der Heijde D, Sharp J, Wassenberg S, et al. Psoriatic arthritis imaging: a review of scoring methods. *Ann Rheum Dis* 2005;64 Suppl 2:ii61-4. doi: 10.1136/ard.2004.030809 [published Online First: 2005/02/15]

2 Pincus T. The American College of Rheumatology (ACR) Core Data Set and derivative "patient only" indices to assess rheumatoid arthritis. *Clin Exp Rheumatol* 2005;23(5 Suppl 39):S109-13. [published Online First: 2005/11/09]
